# Long-term Effects of the Use of a Step Count–Specific Smartphone App on Physical Activity and Weight Loss: Randomized Controlled Clinical Trial

**DOI:** 10.2196/35628

**Published:** 2022-10-24

**Authors:** Eiichi Yoshimura, Eri Tajiri, Ryota Michiwaki, Naoyuki Matsumoto, Yoichi Hatamoto, Shigeho Tanaka

**Affiliations:** 1 Department of Nutrition and Metabolism National Institutes of Biomedical Innovation, Health and Nutrition Tokyo Japan; 2 Graduate School of Environmental & Symbiotic Sciences, Prefectural University of Kumamoto Kumamoto Japan; 3 Faculty of Nutrition, Kagawa Nutrition University Saitama Japan

**Keywords:** step counts, weight loss, smartphone app, step count–specific mobile app, physical activity, moderate-to-vigorous intensity physical activity, lifestyle intervention, mHealth, mobile app: mobile phone

## Abstract

**Background:**

Some studies on weight loss promotion using smartphone apps have shown a weight loss effect but not an increase in physical activity. However, the long-term effects of smartphone apps on weight loss and increasing physical activity have not been rigorously examined to date.

**Objective:**

The aim of this study was to assess whether the use of a smartphone app will increase physical activity and reduce body weight.

**Methods:**

In this parallel randomized clinical trial, participants recruited between April 2018 and June 2019 were randomized in equal proportions to a smartphone app group (n=55) or a control group (n=54). The intention-to-treat approach was used to analyze the data from December 2019 through November 2021. Before the intervention, an hour-long lecture on weight loss instruction and increasing physical activity was conducted once for both groups. Participants in both groups were instructed to weigh themselves immediately after waking up at least once daily from the start of the intervention. Monthly emails were sent advising the participants in both groups on how to lose weight and increase physical activity in order to maintain or increase motivation. Participants in the smartphone app group were instructed to open the app at least once a day to check their step count and rank. The primary outcome was daily accelerometer-measured physical activity (step count) and the secondary outcome was body weight. Since there was a significant difference in the wear time of the accelerometer depending on the intervention period (*P*<.001), the number of steps and moderate-to-vigorous physical activity were also evaluated per wear time.

**Results:**

The mean age of the 109 participants in this study was 47 (SD 8) years. At baseline, the mean daily total steps were 7259 (SD 3256) steps per day for the smartphone app group and 8243 (SD 2815) steps per day for the control group. The difference in the step count per wear time between preintervention and postintervention was significantly different between the app group and the control group (average difference [95% CI], 65 [30 to 101] steps per hour vs –9 [–56 to 39] steps per hour; *P*=.042). The weight loss was –2.2 kg (SD –3.1%) in the smartphone app group and –2.2 kg (SD –3.1%) in the control group, with no significant difference between the groups. In addition, when divided into weekdays (Monday through Friday) and weekends (Saturday and Sunday), there was a significant interaction between step counts (*P*=.004) and MVPA (*P*=.003) during the intervention, with the app group showing higher interaction on weekends than the control group.

**Conclusions:**

In this trial, the group with the smartphone app intervention showed increased physical activity, especially on weekends. However, this increased physical activity did not lead to increased weight loss.

**Trial Registration:**

University Hospital Medical Information Network UMIN000033397; https://center6.umin.ac.jp/cgi-open-bin/ctr_e/ctr_view.cgi?recptno=R000037956

## Introduction

### Background

Physical inactivity–related deaths contribute to US $13.7 billion in productivity losses, and physical inactivity is responsible for 13.4 million disability-adjusted life-years worldwide [1]. Conversely, higher levels of total physical activity at any intensity and less time spent sedentary are associated with a substantially reduced risk of premature mortality [2]. Increasing the amount of physical activity is also effective for weight loss [3,4]. Despite the many health benefits of physical activity, most Japanese adults do not meet the current recommendations for physical activity when assessed using objective measures [5]. Wearing a pedometer encourages increased step count in both the short and long terms [6,7]. A study [8] has shown that those who increased their daily steps over the monitoring period had a substantial reduction in mortality risk. A pedometer-based walking program resulted in moderate weight loss, with participants losing an average of 0.05 kg per week during the intervention period [9].

Recently, the use of mobile apps has led to notable success in increasing physical activity [10,11] and in weight control or weight reduction [12]. In particular, interventions with text messaging and personalization features seem to be more effective [10]. A meta-analysis showed that the use of mobile apps had a positive effect on physical activity measures corresponding to 1850 steps/day [10]. However, a meta-analysis of interventions that promote weight loss by using mobile apps showed a weight loss effect but not an increase in physical activity [12], and several factors might limit the effectiveness of mobile app–based physical activity interventions. First, the duration of most intervention studies using the app is short term (less than 6 months) and the long-term effects of such interventions are still unclear. Second, the 7 papers reviewed in the meta-analysis assessed physical activity by using questionnaires, which may have been unable to assess the change in physical activity. If physical activity can be increased by utilizing mobile apps during weight loss support, it will lead to an increase in energy expenditure, and further weight loss is expected. In addition, to understand the effectiveness of mobile apps in promoting increased physical activity during weight loss support, it is necessary to utilize mobile apps that focus solely on physical activity. Furthermore, body weight and physical activity fluctuate not only seasonally but also within the week [13,14]. Although the effect of app use on physical activity may differ between weekends and weekdays, the difference in the app effects has not yet been elucidated.

### Objective

The purpose of this study was to determine whether using a mobile app would promote increased physical activity and weight loss after 32 weeks of the intervention. In addition, we aimed to assess the intraweek variability of physical activity during the intervention period and to evaluate the impact of using or not using the app.

## Methods

### Study Design and Participants

The participants in this study were recruited using a web portal for municipal employees according to the following inclusion criteria: (1) age, 30-60 years, (2) gained more weight than the weight at 20 years of age, (3) BMI>20 kg/m^2^, and (4) possession of a smartphone. Participants with any disease and who could not obtain permission from their physicians were excluded from the study. The calculated sample size of 102 participants was determined based on a previous study [15] investigating the effect of using a smartphone app on increasing the number of steps taken (effect size=0.564586, α error=.05, power=.80). However, we recruited 110 participants based on the assumption that approximately 10% would drop out, and 109 participants who were finally considered for the analysis were randomized in equal proportions to the smartphone app group (n=55) or control group (n=54) by EY with a random number generator. The participants were first classified by sex and then randomly divided into 2 groups. Recruitment of the target population occurred between April 2018 and June 2019. The 32-week interventions were conducted twice during the same period between June 2018 and January 2020. The last evaluation date was set as 224 days (32 weeks) after the start of the intervention. Assessments of the impact of the intervention on physical activity and body weight were conducted at 10-12 weeks and 30-32 weeks from the start of the intervention. All data analyses were performed at the Kumamoto Prefectural University. The primary and secondary outcomes of this study were step count (physical activity) and body weight.

### Ethics Approval

This study was conducted in accordance with the guidelines of the Consolidated Standards of Reporting Trials (CONSORT). This study followed the guidelines of the Declaration of Helsinki and was approved by the ethics committee for Clinical Research of the Prefectural University of Kumamoto (approval 30-30,01-20) and the ethics committee of the National Institutes of Biomedical Innovation, Health and Nutrition (approval 122-01). Informed consent was obtained from all the participants in this study. The protocol was registered in the University Hospital Medical Information Network (UMIN000033397).

### Intervention

Before the intervention, EY gave both groups 1-hour group-based lectures on weight loss and increasing physical activity. The in-person lecture sessions consisted of 7 domains that focused on the following: (1) the benefits and barriers to engaging in health behaviors, (2) the health benefits of increased physical activity and weight loss, (3) how to calculate energy expenditure by activity intensity, (4) the amount of energy contained in cooked foods and seasonings, (5) how to set a goal of +1000 steps/day (increase walking time by approximately 10 minutes) of increased step count from participants’ current (preintervention period) daily step counts, (6) how to set a weight loss goal of –5% from the participant’s current body weight, and (7) healthy diet and weight maintenance. The participants’ body weight and physical activity were measured for 3 consecutive weeks before and during the intervention (10-12 weeks and 30-32 weeks, respectively). Preintervention evaluations were assessed during the 3 weeks before the intervention began. In addition, dietary intake was assessed during the evaluation period using a questionnaire to assess the average amount of food consumed in the past month. Participants in both groups were instructed to weigh themselves immediately after waking up at least once daily from the start of the intervention. Monthly emails were sent advising the participants in both groups on how to lose weight and to increase physical activity to maintain or increase motivation. In the app group, a smartphone app (present in both Apple and Android smartphones) capable of managing the tracking steps [16] was downloaded by the participants before the intervention. The number of steps taken was displayed in the app in conjunction with the smartphone’s built-in function to evaluate the number of steps taken. The app also displayed the information on walking distance, energy expenditure, and vegetable intake; however, participants were instructed to check only the number and ranking of steps taken. Within the app, the number of steps taken and the rank in the group could be tracked and this information was shared with the smartphone app group. Participants in the app group were instructed to open the app at least once a day to check their step count and rank. Participants in both groups wore accelerometers for 3 weeks that were only set to display the number of steps taken immediately after the intervention began so that they knew how many steps they were taking each day. Participants were asked to compare their daily activity to the feedback results to realize the amount of physical activity that had increased by more than 1000 steps before the intervention. In addition, physical activity and weight data measured prior to the intervention were fed at this time. The results of body weight and physical activity assessments at weeks 10-12 of the intervention were fed back within 1 month; in December, weight change results since the start of the intervention were fed back. The target for the step counts was to increase by at least +1000 steps/day from the preintervention rating. No maximum goal was set and the goal was to reach +1000 steps/day from the preintervention level, and those who reached the goal were encouraged to at least maintain that number of steps. For example, if the average step rating before the intervention was 6000 steps, they were advised to reach at least 7000 steps daily and encouraged to maintain at least 7000 steps even when the goal was reached.

### Anthropometric Measurement

Height was measured using digital scales with a stadiometer to the nearest 0.1 cm (BW-306, Yamato scale) before the intervention period. The body weights of the participants were measured with a body composition monitor to the nearest 50 g (BC-308, Tanita). The participants were instructed to weigh themselves at least once a day, and the time of weighing was to be measured every day within an hour of waking up in the morning, wearing as similar clothes as possible and under fasting conditions. The measured weight and time data were recorded on the Secure Digital card built into the body composition monitor. BMI was calculated as weight (kg) divided by height (in m^2^).

### Physical Activity

Physical activity was measured for 3 weeks [17] at 1-minute epochs by using a triaxial accelerometer (Active Style Pro HJA- 750C) [18] before and during the intervention (10-12 weeks and 30-32 weeks, respectively). The accelerometer was worn on their waist, except while sleeping or bathing. Physical activity was assessed using step count and activity time based on the intensity levels. The obtained physical activity intensity level in each minute was classified as sedentary behavior (≤1.5 metabolic equivalents [METs]), light physical activity (1.6-2.9 METs), and moderate-to-vigorous physical activity (MVPA, ≥3.0 METs). A consecutive zero count of ≥60 minutes was defined as nonwear time. Assessment of accelerometer data was adopted if there was more than 600 minutes of wear time per day. Overall participant wearing time (min/day) of the accelerometer decreased from 940 (SD 102) min/day at baseline to 919 (SD 118) min/day at 12 weeks and 905 (SD 125) min/day at 32 weeks (*P*<.001). Thus, to account for the differences in the wearing time, the average number of steps and MVPA per wearing time (per hour) were calculated with reference to previous studies [11,19]. During the measurement period (before the intervention, 10-12 weeks, and 30-32 weeks), the accelerometer display was set to not be able to see the amount of physical activity for the day.

### Dietary Intake

Food intake was assessed using a validated brief self-administered diet history questionnaire [20,21]. Participants reported the foods they consumed in the past month by selecting the frequency of each food group and the average intake per week. The brief self-administered diet history questionnaire consists of questions on frequency of food and beverage consumption, frequency of rice and miso soup consumption per day, frequency of alcohol consumption and the percentage of alcohol in each of the 5 alcoholic beverages, cooking methods for the dishes eaten most often, and general eating habits. After the participants completed the questionnaire, a dietician checked the completed questionnaire with the participant.

### Statistical Analysis

Changes in the body weight during the intervention are shown as raw data and as a moving average over a week. The demographic variables were assessed using independent sample two-sided *t* tests for continuous data and the chi-square test for categorical data to compare between groups. All analyses were performed using an intention-to-treat approach. To evaluate the effects of using the smartphone app on physical activity and body weight, this study used a mixed-design analysis of variance between the participant groups (smartphone app group vs control group) and within participant groups based on repeated measurements (preintervention vs postintervention at 12 and 32 weeks, respectively). To assess intraweek variability in body weight and physical activity before and during intervention, secondary analyses included comparisons of intraweek changes in body weight and physical activity (step count and MVPA) by using mixed-design analysis of variance. We also estimated the effect of the fixed model and tested its significance for the day of the week with reference to a previous study [17]. The data for body weight are presented in the change based on Friday since the standard error was larger than the intraweek variation and it is difficult to see the variation. Statistical significance was set at *P*<.05. Statistical analyses were performed using the SPSS version 22.0 software (IBM Japan Ltd).

## Results

### Participants and Intervention Adherence

**Figure 1:**
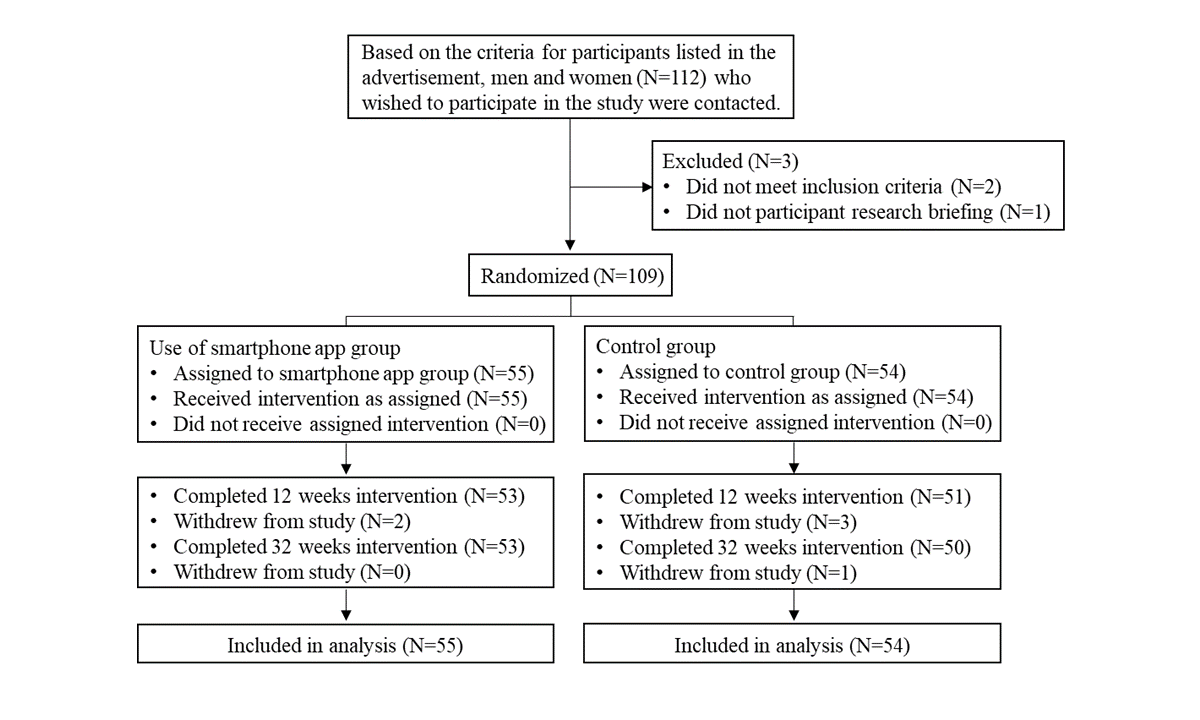
Study flow diagram.

**Figure 2:**
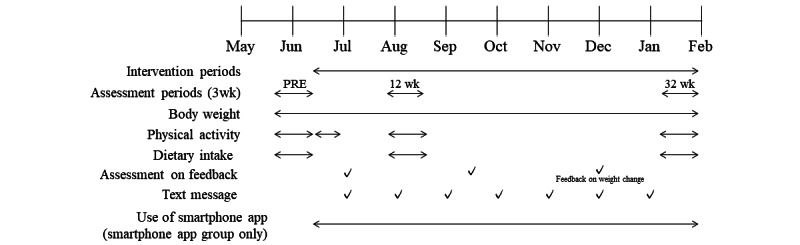
Study protocol. Wk: weeks.

The flowchart of the participants included in this study is shown in Figure 1. The study protocol is illustrated in Figure 2. Two and 3 participants dropped out from the smartphone app and control groups, respectively, between the preintervention and 12 weeks and 1 participant in the control group dropped out at 12-32 weeks. The final dropout rate at 32 weeks was 4% (2/55) in the smartphone app group and 7% (4/54) in the control group, with no significant difference between the 2 groups (*P*=.44). Moreover, before and after the intervention period, 3 participants in the smartphone app group (completion rate, 52/55, 95%) and 6 participants in the control group (completion rate, 48/54, 89%) could not be assessed because the weight and physical activity data were only provided for a few days or due to errors in the analysis process. Thus, the number of participants with complete weight and physical activity data before and after the intervention was 50 in the smartphone app group and 44 in the control group. The proportion of those in the smartphone app group who checked the app at least once a day during the intervention period was 73.4% (overall: 80.1% at the start of intervention to 12 weeks and 73.6% at 12 to 32 weeks). During the 32-week intervention period, the average number of days with at least one weight measurement per day was 187 in the smartphone app group and 185 in the control group. The trends in the weight, BMI, fasting blood glucose level, and hemoglobin A_1c_ level of the 102 participants with the results of physical examinations for the past 10 years and the intervention year are shown in the Multimedia Appendix 1. The year in which the intervention was implemented is shown as zero. The participants tended to gain weight and BMI over the years, and their glycemic status worsened.

### Intervention Effects

**Figure 3:**
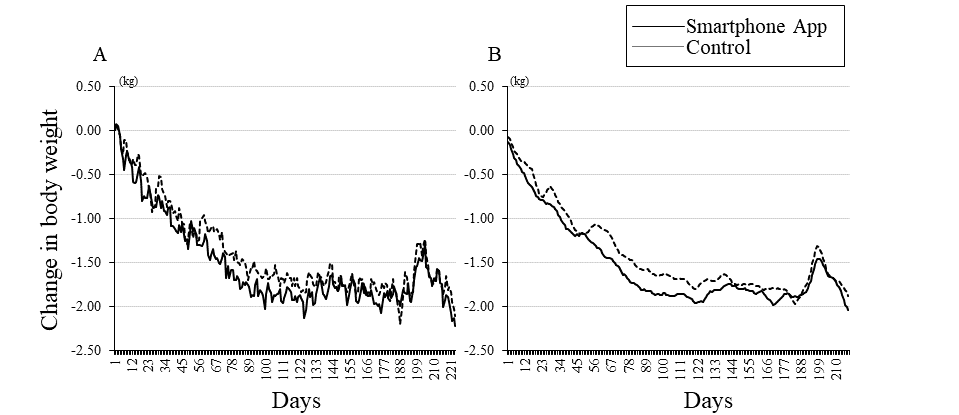
Changes in body weight during the intervention period (32 weeks). The change in the body weight of the participants is shown by the solid line for the smartphone app group and the dotted line for the control group. (A) Values are shown as average and (B) as the moving average over 1 week. The change in body weight before and after the intervention (preintervention, 12 weeks, and 32 weeks) was not significantly different between the groups.

**Figure 4:**
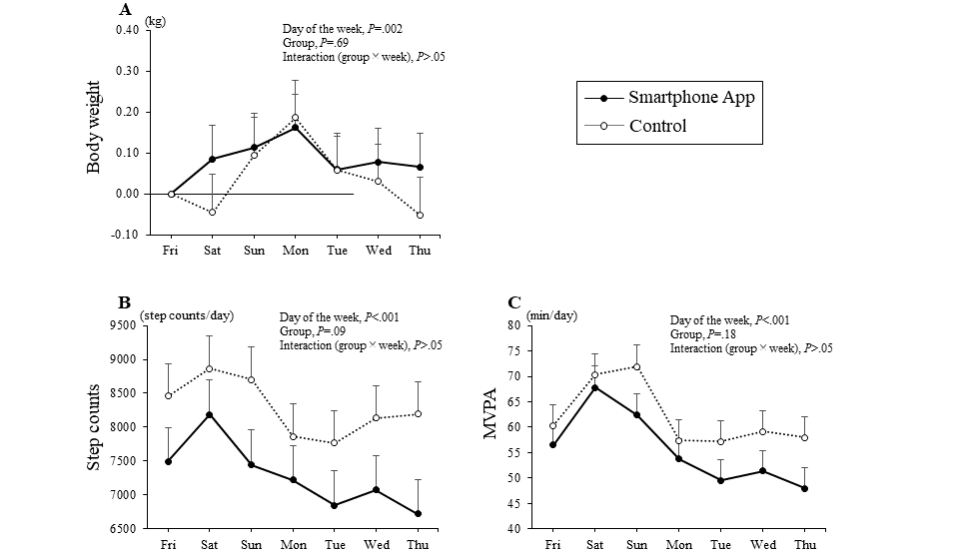
Intraweek variation in (A) body weight, (B) step counts, and (C) moderate-to-vigorous physical activity before the intervention. Missing data were taken into account and analyzed using two-way repeated measures mixed analysis of variance to examine the interaction between the groups. Values are presented as means and standard errors. MVPA: moderate-to-vigorous physical activity.

The baseline data of the 109 participants by their group are shown in Table 1. Figure 3 shows the change in the body weight during the intervention period from June to the end of January (32 weeks) in the years 2018-2020. After 32 weeks of intervention, the weight loss was –2.2 kg (SD –3.1%) in the smartphone app group and –2.2 kg (SD –3.1%) in the control group, with no significant difference between the groups. Intraweek variations in the body weight before the intervention are shown in Figure 4. Before the intervention, there was no significant interaction (group × day of the week) in body weight (Figure 4A), step count (Figure 4B), and MVPA (Figure 4C) between the groups (*P*>.05).

**Figure 5:**
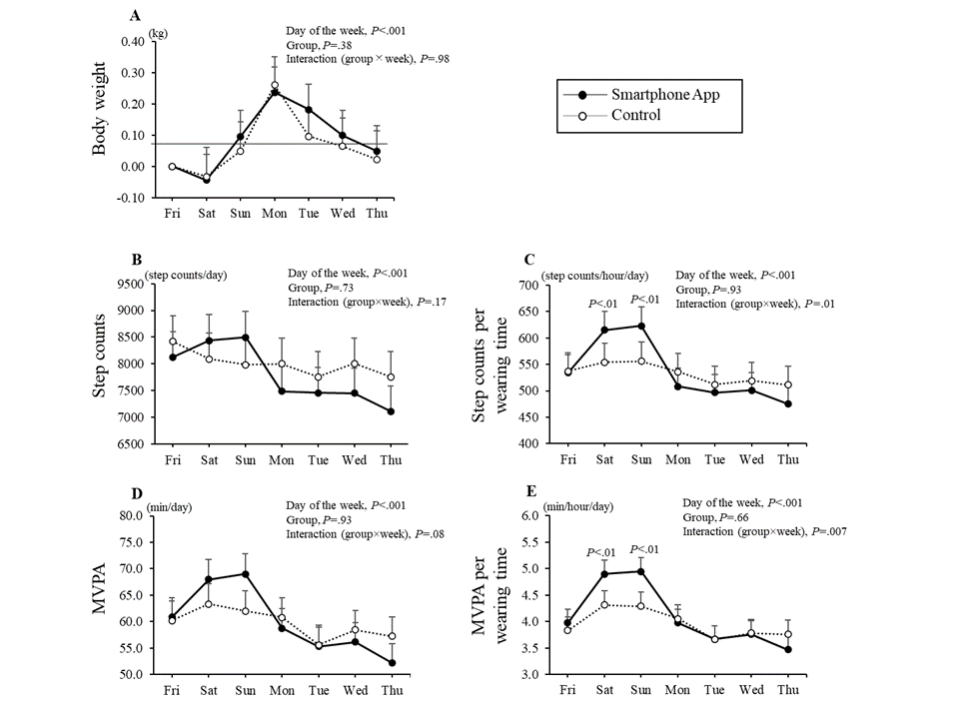
Intraweek variation in (A) body weight, (B and C) step counts, and (D and E) moderate-to-vigorous physical activity during the intervention. Data were combined from 12 and 32 weeks to analyze the relationship between physical activity and intraweek variability. Missing data were taken into account and analyzed using two-way repeated-measures mixed analysis of variance to examine the interaction between the groups. Values are presented as means and standard errors. MVPA: moderate-to-vigorous physical activity.

The effects of the intervention on body weight and physical activity before and after the intervention are shown in Table 2. The step count per wear time and MVPA per wear time showed a significant interaction between the groups (*P*=.04 and *P*=.03, respectively). Similar results were obtained for the per-protocol set. The intraweek variations in body weight, step count, and MVPA with or without wear time during the intervention period are shown in Figure 5. There was a significant interaction (group × day of the week) in the step count and MVPA per wear time between the groups (*P*=.01 and *P*=.007, respectively), and the step counts on Saturdays and Sundays in the smartphone app group were higher than those in the control group (*P*<.05). In addition, when divided into weekdays (Monday through Friday) and weekends (Saturday and Sunday), there was a significant interaction between step counts (*P*=.004) and MVPA (*P*=.003) during the intervention, with the app group showing higher interaction on weekends than the control group. In the analysis including both groups, the correlations between change in step counts and change in energy intake (*r*=–0.141) and weight loss (*r*=–0.026) were not statistically significant; however, there was a significant correlation between dietary intake and weight loss (*r*=0.198, *P*=.048).

**Table 1:** Baseline information of the participants (N=109).

			Smartphone app group (n=55)		Control group (n=54)		*P* value
**General characteristics**							
	Women, n (%)	26 (47)		24 (44)		.85	
	Age (years), mean (SD)	47 (8)		47 (8)		.93	
	Body weight (kg), mean (SD)	71.0 (13.9)		70.0 (13.0)		.69	
	BMI (kg/m^2^), mean (SD)	25.9 (4.1)		25.3 (3.6)		.46	
	Step counts (steps/day), mean (SD)	7259 (3256)		8243 (2815)		.09	
	Activity time of 1.5 METs^a^ (min/day), mean (SD)	571.5 (99.6)		603.5 (109.5)		.11	
	Activity time of 1.6-2.9 METs (min/day), mean (SD)	302.2 (79.9)		289.3 (69.6)		.37	
	Activity time of over 3.0 METs (min/day), mean (SD)	55.2 (25.2)		61.5 (23.8)		.18	
	Activity time of 1.5 METs (%), mean (SD)	61.4 (8.7)		62.9 (6.6)		.31	
	Activity time of 1.6-2.9 METs (%), mean (SD)	32.7 (8.0)		30.5 (6.2)		.13	
	Activity time of over 3.0 METs (%), mean (SD)	6.0 (2.7)		6.6 (2.6)		.25	
	Energy intake (kcal/day), mean (SD)	2005 (649)		1857 (624)		.23	
	Protein (%), mean (SD)	14.6 (2.9)		15.5 (2.6)		.08	
	Fat (%), mean (SD)	27.8 (5.1)		28.9 (5.6)		.29	
	Carbohydrate (%), mean (SD)	57.6 (7.1)		55.5 (7.6)		.16	
**Self-reported cardiovascular risk factors, n (%)**							
	Overweight/obese^b^	25 (45)		22 (41)		.70	
	Obese^c^	8 (15)		7 (13)		>.99	
	Hypertension	5 (9)		6 (11)		.76	
	Dyslipidemia	1 (2)		4 (7)		.21	
	Diabetes	1 (2)		0 (0)		>.99	
	Current smoker	2 (4)		5 (9)		.27	

^a^MET: metabolic equivalent.

^b^BMI≥25 kg/m^2^.

^c^BMI≥30 kg/m^2^.

**Table 2:** Intervention effects on body weight, physical activity, and dietary intake before and after intervention.

			Smartphone app group							Control group							Group × interaction, *P* value
			Preintervention, mean (95% CI)		12 weeks, mean (95% CI)		32 weeks, mean (95% CI)		Preintervention, mean (95% CI)			12 weeks, mean (95% CI)		32 weeks, mean (95% CI)			
**Intention-to-treat**																	
	Body weight (kg)	70.9 (67.4-74.5)		68.8 (65.3-72.4)		68.8 (65.2-72.3)		70.0 (66.4-73.5)			68.0 (64.4-71.6)		67.8 (64.2-71.4)		.94		
	Step count (steps/day)	7259 (6335-8183)		7850 (6921-8779)		7846 (6910-8782)		8243 (7465-9021)			8143 (7346-8940)		7806 (6998-8615)		.06		
	Step counts per wear time (steps/h/day)	473 (408-538)		527 (462-592)		538 (473-604)		525 (472-579)			528 (4737-583)		517 (461-572)		.04		
	MVPA^a^ (min/day)	55 (48-62)		58 (51-65)		62 (55-69)		62 (55-68)			60 (54-67)		59 (52-65)		.05		
	MVPA per wear time (min/h/day)	4 (3-4)		4 (3-4)		4 (4-5)		4 (3-4)			4 (3-4)		4 (3-4)		.03		
	Energy intake (kcal/day)	2005 (1844-2166)		1783 (1621-1946)		1836 (1673-2000)		1857 (1694-2020)			1718 (1553-1883)		1758 (1593-1923)		.66		
	Protein (%)	14.6 (13.8-15.4)		15.6 (14.8-16.4)		15.5 (14.7-16.3)		15.5 (14.7-16.3)			16.1 (15.3-16.9)		16.1 (15.3-16.9)		.65		
	Fat (%)	27.8 (26.3-29.4)		28.7 (27.1-30.2)		27.5 (25.9-29.1)		28.9 (27.3-30.5)			29.5 (27.9-31.1)		28.3 (26.7-29.9)		.96		
	Carbohydrate (%)	57.6 (55.4-59.7)		55.7 (53.5-57.9)		57 (54.8-59.2)		55.5 (53.4-57.7)			54.4 (52.2-56.6)		55.7 (53.4-57.9)		.87		
**Per protocol**																	
	Body weight (kg)	70.6 (67.0-74.2)		68.4 (64.9-71.9)		68.4 (64.9-71.9)		68.3 (64.7-72.0)			66.4 (62.8-69.9)		66.2 (62.6-69.7)		.90		
	Step count (steps/day)	7130 (6287-7972)		7833 (6939-8728)		7779 (6874-8685)		8231 (7333-9129)			8149 (7196-9103)		7826 (6861-8791)		.05		
	Step counts per wear time (steps/h/day)	465 (408-522)		527 (465-588)		535 (467-602)		526 (465-587)			527 (462-592)		520 (448-592)		.047		
	MVPA (min/day)	54 (48-61)		58 (51-65)		61 (54-68)		61 (54-68)			60 (53-67)		59 (51-66)		.04		
	MVPA per wear time (min/h/day)	4 (3-4)		4 (3-4)		4 (4-5)		4 (3-4)			4 (3-4)		4 (3-4)		.03		
	Energy intake (kcal/day)	1966 (1800-2133)		1759 (1600-1919)		1811 (1677-1945)		1851 (1680-2022)			1704 (1539-1870)		1752 (1576-1927)		.79		
	Protein (%)	14.8 (14.0-15.5)		15.7 (14.9-16.6)		15.6 (14.8-16.4)		15.7 (15.0-16.4)			16.2 (15.4-17.1)		16.2 (15.3-17.0)		.68		
	Fat (%)	27.8 (26.4-29.2)		28.7 (27.1-30.3)		27.5 (25.8-29.2)		28.8 (27.2-30.3)			29.3 (27.6-30.9)		28.0 (26.3-29.7)		.90		
	Carbohydrate (%)	57.4 (55.4-59.4)		55.5 (53.2-57.9)		56.9 (54.5-59.2)		55.5 (53.4-57.6)			54.5 (52.2-56.8)		55.9 (53.5-58.2)		.81		

^a^MVPA: moderate-to-vigorous physical activity.

## Discussion

The aim of this study was to determine the effects of using a mobile app on increasing physical activity and weight loss by assessing accelerometer data and weight loss data after 32 weeks of the app intervention. Our findings showed that the use of a step count–specific mobile app for the assessment of physical activity for weight loss might be effective in increasing the step count, although it may not affect the amount of weight loss. In addition, we found that the effects of using the mobile app on physical activity differed between weekends and weekdays and that the mobile app showed data of higher physical activity on weekends.

Flores Mateo et al [22] and Islam et al [12] reported meta-analyses that were related to our study. Their meta-analyses indicated that the app intervention group showed more weight loss than the control group, but there was no statistically significant change in physical activity. Most previous studies assessed habitual physical activity through questionnaires or self-reports [15,23-28]. To our knowledge, our study is the first to assess physical activity by using accelerometers for weight loss and to examine the long-term effects of physical activity by using step count–specific mobile apps. In this study, we found that the use of a step count–specific mobile app for weight loss led to an increase in physical activity. Previous studies have demonstrated that increased physical activity reduces the risk of mortality and has various physiological benefits [29-31]. The impact on physical activity from using a step-counting app for weight loss is not as significant as expected; however, continued use might provide physiological benefits. In the future, the long-term impact of using health indicators should be examined.

Inconsistent with that reported in a meta-analysis [22], the change in weight loss was not significantly different between the 2 groups in our study. This may be because the control group in our study demonstrated a certain weight loss effect after 1 lecture and a few text messages even without using the app. A study has shown that a single motivational lecture can promote moderate weight loss in the short term [32]. Moreover, our study and the previous study [32] consisted of populations that were motivated to lose weight, which may have influenced the results. In addition, many intervention studies using mobile apps included several support tools in the apps, such as texts, emails, internet, interactive chatbots, and voice agents. Although the effectiveness of an intervention may be increased by the inclusion of many support features in mobile apps, it is difficult to understand the exact factors that affect the observed changes. Pedometers and accelerometers affect the number of steps just by wearing them, but it is unclear whether the use of mobile apps that focus only on step count affects the step count. If the number of steps taken by a 70-kg person increases by 1000 and the intensity is 3 METs, the person should lose more than 1 kg in 32 weeks, but our study showed no such effect. Wu et al [33] indicated in their meta-analysis that the pooled weight loss was 1.14 kg or 0.50 kg/m^2^ greater for the diet-plus-exercise group than for the diet-only group. However, results with an intervention period of less than 1 year showed no intervention effect. Furthermore, most studies did not show the expected weight loss effect from the energy expenditure generated by the set exercise [33-35]. Interventions that combine dietary restriction and physical activity may attenuate the effects of physical activity interventions. The weight loss after 32 weeks of the intervention in our study was approximately 2 kg. Further improvements in lecture content and support tools are needed to increase the effectiveness of additional weight loss. In addition, participants in the app group were instructed to use the app daily to check their steps and rank. Although such an approach is intended to motivate participants, we could not assess the motivational impact of app use. Further research is needed in this direction in the future.

Notably, we found that the effects of the mobile app on physical activity differed between weekends and weekdays, that is, the mobile app data showed higher physical activity on weekends. In a study on Japanese white-collar workers, the sedentary behavior time was significantly longer on weekdays than on weekends (598 min/day vs 479 min/day, respectively; *P*<.001) [36]. However, among blue-collar workers, there was no significant difference in the sedentary behavior time between weekdays and weekends (462 min/day vs 485 min/day; *P*=.43) [36]. The proportion of workers who achieved the recommended sedentary behavior levels (≤8 hours) was only 4.8% for white-collar workers on weekdays and 54.8% on weekends (*P*=.04) [36]. All the participants in our study were prefectural employees, and the weekends were Saturday and Sunday. Although change is required regarding work or after-work behavior on weekdays, it may be difficult to increase the number of steps taken on weekdays. Our study showed that the use of smartphone apps could increase physical activity by increasing the step count, especially during leisure time on nonworking days such as Saturday and Sunday. These results can be obtained using an accelerometer. The originality of this study is in the use of accelerometers to assess physical activity and the use of up to 6 weeks of physical activity data assessed at weeks 12 and 32 of the intervention to examine the within-week variability.

The dropout rate in our study was less than 10%, despite the long intervention period of 32 weeks, and there was no difference between the 2 groups. The reasons for this cannot be ascertained; however, intervention content such as monthly emails and feedback on the results may have had an impact. Continued participation in the intervention is an essential factor affecting the validation of intervention effectiveness and should be investigated in future studies.

This study has several limitations. First, all the participants in this study were prefectural employees, which limits the generalizability of the study. Second, although this study calculated the sample size and conducted an intervention, it is possible that sample size estimation was inadequate due to the lack of appropriate studies utilizing apps focused on step count and using physical activity and weight loss as outcomes. Future large-scale intervention studies are needed. Third, although the duration of the intervention in this study was longer compared to that in previous studies, it is necessary to examine the impact of the intervention for more than 1 year, and the influence of the seasons needs to be considered. Further, the use of the app may have affected the motivation for the intervention but could not be assessed in this study. Future studies should also evaluate the motivational impact of app use. Finally, it is necessary to develop support tools to increase not only the amount of physical activity but also the weight loss effect. However, the major strength of our study was the parallel randomized controlled trial design, which indicates that our findings are reliable.

In conclusion, this study shows that the use of a step count–specific mobile app during weight loss support might be effective in increasing the step count, although it might not affect the amount of weight loss. Moreover, we found that the effects of the mobile app on physical activity differed between weekends and weekdays, with the mobile app data showing higher physical activity on weekends. Future studies need to focus on the development of methods for increasing the effectiveness of physical activity and weight loss by using mobile apps.

## Appendix

Multimedia Appendix 1 Changes in body composition and blood glucose levels in physical examination findings. Missing data were considered and analyzed using one-way repeated-measures mixed analysis of variance. Values are presented as means and standard errors.

Multimedia Appendix 2 CONSORT-eHEALTH (V 1.6.1) checklist.

## Abbreviations

CONSORT: Consolidated Standards of Reporting Trials
MET: metabolic equivalent
MVPA: moderate-to-vigorous physical activity
