# Supplementary figures and images for: Long-term Effects of the Use of a Step Count–Specific Smartphone App on Physical Activity and Weight Loss: Randomized Controlled Clinical Trial
Source: JMIR Mhealth Uhealth. 2022 Oct 24;10(10):e35628. doi: 10.2196/35628 (PMC9641518; doi:10.2196/35628)

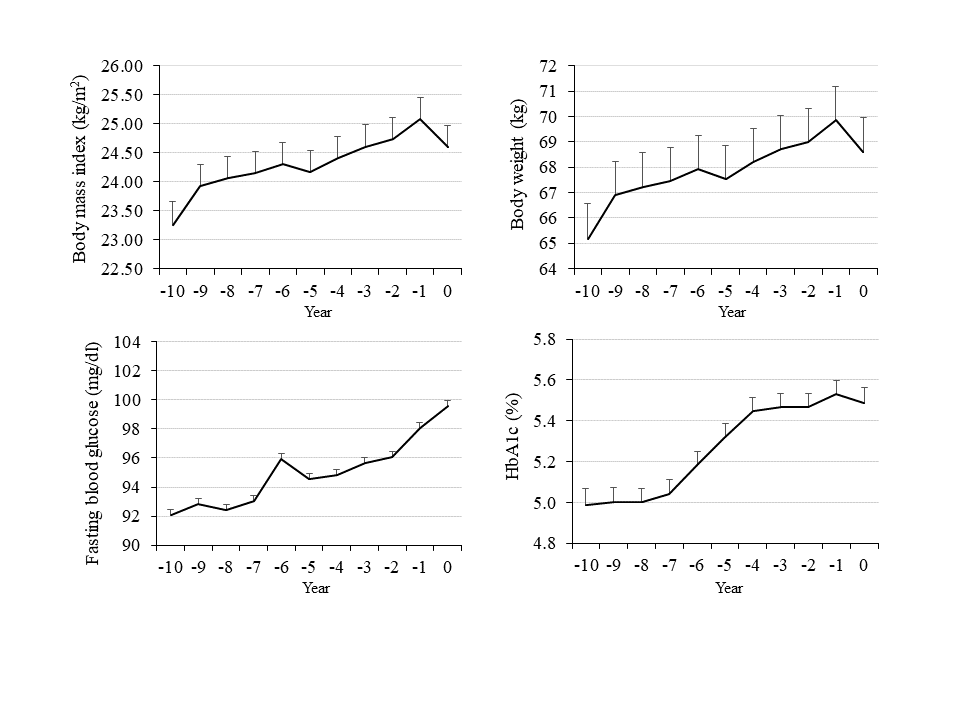

Supplement: Multimedia Appendix 1 [file mhealth_v10i10e35628_app1.png]
